# Empirical Models of Demand for Out-Patient Physician Services and Their Relevance to the Assessment of Patient Payment Policies: A Critical Review of the Literature

**DOI:** 10.3390/ijerph7062708

**Published:** 2010-06-23

**Authors:** Olga Skriabikova, Milena Pavlova, Wim Groot

**Affiliations:** 1 Department of Health Organization, Policy and Economics, CAPHRI, Medical Center, Faculty of Health, Medicine and Life Sciences, Maastricht University, P.O. Box 616, 6200 MD Maastricht, The Netherlands; E-Mails: m.pavlova@BEOZ.unimaas.nl (M.P.); w.groot@maastrichtuniversity.nl (W.G.); 2 Research Center for Education and the Labour Market (ROA), School of Business and Economics, Maastricht University, P.O. Box 616, 6200 MD Maastricht, The Netherlands; 3 Topinstitute Evidence Based Education Research (TIER), Maastricht University, P.O. Box 616, 6200 MD Maastricht, The Netherlands

**Keywords:** demand modeling, out-patient physician services, patient payments, policy analysis

## Abstract

This paper reviews the existing empirical micro-level models of demand for out-patient physician services where the size of patient payment is included either directly as an independent variable (when a flat-rate co-payment fee) or indirectly as a level of deductibles and/or co-insurance defined by the insurance coverage. The paper also discusses the relevance of these models for the assessment of patient payment policies. For this purpose, a systematic literature review is carried out. In total, 46 relevant publications were identified. These publications are classified into categories based on their general approach to demand modeling, specifications of data collection, data analysis, and main empirical findings. The analysis indicates a rising research interest in the empirical micro-level models of demand for out-patient physician services that incorporate the size of patient payment. Overall, the size of patient payments, consumer socio-economic and demographic features, and quality of services provided emerge as important determinants of demand for out-patient physician services. However, there is a great variety in the modeling approaches and inconsistencies in the findings regarding the impact of price on demand for out-patient physician services. Hitherto, the empirical research fails to offer policy-makers a clear strategy on how to develop a country-specific model of demand for out-patient physician services suitable for the assessment of patient payment policies in their countries. In particular, theoretically important factors, such as provider behavior, consumer attitudes, experience and culture, and informal patient payments, are not considered. Although we recognize that it is difficult to measure these factors and to incorporate them in the demand models, it is apparent that there is a gap in research for the construction of effective patient payment schemes.

## 1. Introduction

The increase in the demand for health care services combined with a lack of public resources have resulted in attempts of governments to explore different methods of health care financing, including various forms of patient payments [1]. There are two main expectations related to the implementation of patient payments. On the one hand, patient payments are seen as a policy tool that can help to control service utilization. It is expected that patients who pay fees are likely to become more cost-conscious and to seek only services that they really need [2]. On the other hand, fees charged from patients are a source of additional revenue for the health care sector [3]. It is expected that patient payments raise additional resources for the expansion of health care provision and the improvement of service quality [4].

In order to construct effective and equitable payment schemes, policy-makers need scientific evidence on the effects of patient payments on consumer behavior, and more generally, on the demand for health care in a given context [1]. Lack of understanding of consumer demand for health care and its determinants could lead to the implementation of patient payment schemes that are ‘catastrophic’ for the population [5]. This necessitates an empirical analysis of consumer demand for health care prior to the implementation or amendment of patient payment schemes in a country.

Such preliminary analysis would be possible however, if among other things, policy-makers are provided with a country-specific model of demand for health care suitable for the assessment of patient payment policies. This demand model needs to account for factors related to consumer behavior (e.g., consumer preferences and willingness to pay, as well as attitudes, experience and culture) under alternative patient payment schemes [6,7]. In addition to this, supply-side factors (e.g., provider reimbursement schemes and reallocation of patient payments collected) also have to be considered. If health care providers are involved in the process of fee collection, the introduction of patient payments can affect their behavior resulting in excess supplier-induced demand. Other relevant factors are the existence and prices of alternative health care services (e.g., in the private sector), as well as the existence and levels of informal patient payments. Informal payments include both unofficial cash payments (also called under-the-table payments), and gifts in kind requested or expected by medical staff for proving medical services with better quality and quicker access, or in some instances, for providing medical services in general [8,9]. In some countries, official patient payments have imposed a double financial burden on consumers because they have been implemented in a context of persistent informal payments for health care services [10,11].

This paper focuses on out-patient physician services. In particular, the paper systematically reviews the existing empirical micro-level models of demand for out-patient physician services where the size of patient payment is included either directly as an independent variable (when a flat-rate co-payment fee) or indirectly as a level of deductibles and/or co-insurance defined by the insurance coverage. The paper also analyzes the relevance of these models for the analysis of patient payment policies. The following two sections of the paper describe the methods used for the systematic literature search and the main characteristics of the empirical demand models identified in this search. The next section presents an interpretation and discussion of the findings. Finally, the paper outlines recommendations for policy and research.

## 2. Materials and Methods

Three main keywords were selected for the systematic literature review, namely DEMAND, MODEL and PATIENT PAYMENT. In addition to this, synonyms of the above-mentioned keywords were added. Differences in spelling were also considered. This resulted in the following final chain of keywords that was used for the literature search: DEMAND and MODEL or MODELLING or MODELING and PATIENT PAYMENT or USER FEE or USER CHARGE or COST-SHARING or DEDUCTIBLE or CO-PAYMENT or COPAYMENT or CO-INSURANCE or COINSURANCE or OUT-OF-POCKET PAYMENT or INFORMAL PAYMENT. Additional keywords, namely PATIENT and HEALTH, were added to focus the search on the health care context. In order to include all possible word forms, a truncation function was used.

The search for relevant publications with the chain of key-words presented above was conducted in PubMed, EconPapers, Medline, Embase and JSTOR. Based on the initial literature search, a primary list of publications was obtained.

The list was reduced using a set of limitation and relevance criteria. Specifically, the language of the publication was limited to English and the type of publication was limited to peer-reviewed journal articles. There was no limitation with regard to the year of publication.

The first relevance criterion concerned the focus of the study reported in a publication. Only publications that presented empirical micro-level analysis of demand for out-patient physician services were selected as relevant. Thus, publications that analyzed demand for inpatient, dental, mental, emergency, preventative and long-term care, as well as demand for pharmaceuticals and alternative medicine, were excluded. For the same reason, the review excluded publications that analyzed demand for health insurance, health care and/or health in general where the demand for physician services was not modeled separately. Also, publications that focused solely on the use of physician services, choice of provider and willingness-to-pay for physician services with no explicit demand modeling were also excluded. Since the review was focused on empirical micro-level demand modeling, publications that had only qualitative evaluations of demand or analyze aggregated population demand, as well as editorials and secondary analyses (e.g., reviews) were not included.

Publications that met the first relevance criterion were evaluated based on the second relevance criterion concerning the inclusion of the size of patient payments in the analysis. Specifically, the final list included only publications that present a demand model where the exact size of patient payment was included either directly as an independent variable (when a flat-rate co-payment fee) or indirectly as an exact level of deductibles and/or co-insurance defined by the insurance coverage. As a result, publications that did not include the exact patient payment size but for example, only non-monetary price, opportunity costs and/or travel costs, or only an indicator of low/high patient payments, or no price at all, were excluded from the review.

The completeness of the final list of relevant publications was checked based on a review of the reference lists of publications identified as relevant and based on suggestions by experts in the field.

The publications selected as relevant were reviewed to identify the main characteristics of the empirical micro-level models of demand for out-patient physician services. The study designs, and the methodology used to obtain the empirical data, as well as the main empirical findings were also reviewed (even though the latter is not the main objective of this review). The results of the review were summarized in the form of tables that comprise categories related to: (1) general characteristics of the studies; (2) specifications of the demand modeling process; (3) methods of data collection and analysis; (4) major research findings. These groups of categories were taken into account to interpret the results of the literature review in view of the assessment of patient payments.

## 3. Results and Discussion

An initial list of 468 publications was obtained. In total 46 publications that meet the limitation and relevance criteria were included in the literature review. The results of the review are presented in this section in the form of tables which have a similar structure throughout the paper. The first column of each table provides a set of categories used to classify the publications. In the second column, the number and percentage of publications that fall into a given category is presented. The third column indicates the number of a given publication in the reference list.

### 3.1. General Characteristics of the Selected Publications

The overall characteristics of the publications included in the review are presented in Table 1. As indicated in this table, the majority of publications are recently published (since 1995). Most publications report empirical analysis of demand for physician services in the USA (labeled as North America in Table 1), although a number of demand studies from European, African and Asian countries are also included in the review. Cross-country comparisons are lacking. The majority of the publications report data collected in household settings. Health care organizations are rarely used as a research setting.

As indicated in Table 1, more than half of all publications directly aim to assess the impact of patient payments on the demand for out-patient physician services. The rest of the publications state that their direct aim is either to model demand for or use of out-patient physician services in general, or to analyze the impact of health insurance on the use of these services. Modeling the choice of physicians is seldom stated as a direct research aim.

### 3.2. Specificity of the Data Collection Process

A summary of the data collection process reported in the 46 publications is provided in Table 2. The table suggests that the vast majority of publications use data collected in cross-sectional surveys and only few publications report experimental designs, such as controlled or natural experiments. In about half of the publications, sample selection is based on specific population characteristics because authors use existing datasets (e.g., from national surveys) and extract the sample according to research interests in specific population group. Nine publications specify that they used a random sample selection and another ten publications use stratified or cluster random selection.

Table 2 also suggests that the sample size is usually large, *i.e.*, more than 1,000 or even more than 10,000 respondents. However, the response rate is rarely reported. Only a few publications provide information about the response rate and analyze/discuss potential biases due to a selective non-response rate. When the publications report own data collection, the most common methods applied are either interviews or questionnaires.

### 3.3. Specificity of the Data Analysis

The characteristics of the data analysis and demand modeling reported in the selected publications are presented in Table 3. Almost all publications included in the review are based on a revealed preferences approach (*i.e.*, data about past consumer behavior). Only one publication reports a stated preferences approach based on statements of consumers about their hypothetical (future) behavior.

More than half of the publications take the probability of visiting a physician or the number of visits to physician as a dependent variable. In all publications, the size of patient payments is included either as an independent variable (in case of a flat-rate co-payment fee) or indirectly as a level of deductibles and/or co-insurance, since this was one of the relevance criteria for the selection of publications for this review. In total 19 publications report models where other (indirect) payments (e.g., travelling and waiting time) are also taken into account. Consumer demographic and socio-economic features, as well as health status are included as independent (explanatory) variables in the majority of the publications. One third of the publications incorporate provider characteristics, such as quality and location.

As indicated in Table 3, most publications use a type of non-linear regression for data analysis (e.g., a type of logit, probit, tobit or count data regression). Linear (*i.e.*, ordinary least-square) regression is relatively less often used. In some cases, two-part or hurdle models are employed, with the first stage formed by the decision to see a physician (e.g., by probit or logit regression) while in the second stage, the number of visits to the physician is analyzed (e.g., by least square regression or count data specification). About a quarter of all publications report simulation methods as an analytical technique. Most publications report other statistical results, such as descriptive statistics, as well as goodness-of-fit statistics.

### 3.4. Major Findings Reported in the Selected Publications

The main findings of the empirical demand analyses reported in the 46 publications as well as the discussion of their reliability, validity and generalizability are summarized in Table 4. More than half of the publications find that the impact of price (*i.e.*, the patient payment level) on the quantity of out-patient physician services demanded is statistically significant. The price elasticity is reported in two thirds of the publications. Its most usual value is less than 0.50, *i.e.*, relatively low price elasticity. However, 3 publications that report demand analyses for developing countries, indicate higher price elasticity: between 0.50 and 1, and/or higher than 1.

More than half of the publications report statistically significant determinants of the price elasticity of demand for out-patient physician services. Consumer income is overall the most important factor influencing the price elasticity, followed by characteristics of the provider (such as quality and location) and the availability of alternative health care services. One publication reports that the nonmonetary price factors are also significant determinants of price elasticity. Some publications report that price elasticity depends on the price range, *i.e.*, it is higher at the lower price levels and *vice versa*.

In addition to price, several other factors emerge as important determinants of the demand for out-patient physician services in most or several publications. These include consumers’ socio-economic and demographic characteristics (e.g., age, gender, education, income), time-related payments by consumers, quality and location of service provision, and the price of alternative health care services.

At the bottom of Table 4, the publications are classified according to the validity, reliability and generalizability of the results. A study is considered reliable if the methods of data collection and analysis are well defined and potentially repeatable, and when multiple models or multiple samples produce comparable results. Based on this definition, 19 publications among all publications clearly state the methods of data collection and analysis used, and thus, present a reliable demand analysis. The reliability of the analyses in the rest of the publications is uncertain. Measures of validity included the level of consistence of the obtained results with the stated study hypothesis (theoretical validity), convergence with the results reported in other publications (convergent validity) and indicated attempts to address predictive power of the results (predictive validity). Based on these criteria, 37% of the publications report evidence that confirms certain aspects of validity, namely theoretical, convergent or predictive validity of the findings. Nevertheless, predictive validity is a relatively infrequent subject of discussion. The generalizability of the results is clear in 15% of the publications.

## 4. Discussion

The results of the systematic literature review reported in the previous section indicate a rising research interest in empirical micro-level models of demand for out-patient physician services where the size of patient payment is included either directly as an independent variable (when a flat-rate co-payment fee) or indirectly as a level of deductibles and/or co-insurance defined by the insurance coverage. However, this research is not solely driven by a direct interest in the impact of patient payment schemes. The empirical models reviewed in this paper are also developed to identify relevant determinants of demand for and use of out-patient physician services. Such determinants are important for example when identifying tools for controlling the health care expenditures, which is also a highly relevant policy question [50,58].

### 4.1. The Approach to Modeling

The prevailing approach in modeling demand for out-patient physician services is the revealed preferences approach using data on past consumer behavior. This is probably because the majority of publications reviewed, present analyses based on existing datasets where stated preference data are not available. Utilization of existing datasets is comparatively inexpensive and less time-consuming, but the datasets might lack relevant information (e.g., on social and health characteristics of the study population) that limits the validity of the results. Another consequence of using existing datasets is that it is impossible to estimate response rates and analyze the reasons for non-response together with its potential bias on the interpretation of the results. Additionally, ex-post evaluation of consumer behavior does not provide exact information about the motivation of this behavior and therefore, it does not allow tracking all demand determinants [59]. Thus, causal relations between patient payment policies and the behavioral responses of consumer are difficult to establish.

The stated preference approach to the analysis of demand for out-patient physician services could help to overcome these pitfalls but so far, is rarely applied. The techniques for collecting stated preference data (e.g., discrete-choice experiment and contingent valuation) are relatively new and they are often criticized for their potentially low predictive validity [60–62]. This could explain why we found only one application of the stated preference approach for modeling demand for out-patient physician services. However, the stated preference approach has an important strength. It allows experimentation with policy designs (including patient payment policy) without the need to implement or change this policy. In some instances, actual experimentation with different policy designs might be unethical and socially unacceptable (e.g., experimentation with expansion and reduction of fee magnitudes), or might be impossible if policy is still not implemented. Therefore, it is not surprising that we found only few publications that report experimental designs for data collection.

Even when actual experimentation is possible, data on actual consumer behavior might be interwoven with problems related to co-variation between service attributes (e.g., price and quality) as well as problems related to self-selection and generalization [63]. Individuals who decide for a certain type of behavior are not the random sample of the entire population. Although there are methods to account and correct for the self-selection bias, none of them is completely satisfactory and some of them lead to outcomes that are decidedly disputable [63]. These limitations to the actual experimentation with policy design necessitate the application of stated preference techniques in empirical demand modeling. Furthermore, exploration of mixed approaches, *i.e.*, the combination of stated and revealed preferences methods, to compare actual consumer behavior with statements about this behavior (see [64]) is of particular importance for demand modeling. For example, a mixed approach may be used where a population survey that contains stated preferences questions is followed by a validation experimental stage where a part of the population is included.

### 4.2. Variables Included in Demand Models

The majority of empirical models of demand for out-patient physician services reviewed in this paper, account for relevant determinants of demand, such as socio-demographic characteristics of consumers, direct and indirect (e.g., time-related) payments by consumers, some providers characteristics (such as quality and location), and the price of alternative health care services. However, we did not find studies that investigate in detail the possible influence of provider behavior on consumer demand for out-patient physician services. In particular, supply-side factors relevant to the evaluation of patient payment polices (such as the impact of provider reimbursement schemes and the involvement of providers in the collection of patient payments) are not included in the empirical demand models reviewed. Some publications (e.g., [52]) discuss the possibility of supplier-induced demand as a result of the provider reimbursement scheme applied. Nevertheless, the empirical models of demand presented in these and other publications are based on data from a single country where most supply-side factors lack variability and cannot be analyzed.

In addition to this, not all consumer and patient payment characteristics relevant to the evaluation of patient payment policies are considered in the empirical models of demand reviewed. For example, some consumer-specific factors (such as attitudes, experience and culture), which effect on demand is broadly recognized [6,7], are overall lacking in these demand models. Furthermore, informal patient payments are not considered in any of the publications reviewed. The inclusion of these additional consumer and patient payment characteristics in demand analyses can be crucial because cultural and attitudinal differences among consumers, as well as the level of informal patient payments, may cause different reactions to changes in the patient payment schemes [65]. While from a theoretical point of view these might be important determinants of demand, such data are usually not readily available in existing datasets (as discussed above) or are difficult to collect in case of informal payments [8].

### 4.3. The Empirical Results

Although the analysis of the empirical results reported in the publications that we reviewed, is not the primary objective of this paper, the overview of these results (see Table 4) suggests some relevant discussion points, namely with regard to price elasticity and its determinants, and other non-price determinants of demand.

Overall, the size of patient payments emerges as an important determinant of demand for out-patient physician services. However, there is a great variety in modeling approaches and inconsistencies of findings regarding the impact of price. In general, the price-elasticity of demand for out-patient physician services is higher in case of lower income, higher patient payments and when alternative health care services exist. Some publications indicate that price significantly influences the quantity of services demanded, while others find this influence statistically insignificant. Moreover, the comparison of the results shows that the values of the price-elasticity reported are rather controversial ranging from very high (higher than 1) to very low (lower than 0.10). This could be explained by the considerable differences in settings, study populations and analytical methods used in the demand analyses. Empirical demand analyses in other areas of the health care sector (namely hospital services) also indicate that the analytical method could be an essential factor when analyzing price-elasticity [36]. Moreover, only few publications report high price elasticity (higher than 0.5) and they are based on data from low-income countries, which may explain the difference with price-elasticity reported in other studies. It should be also acknowledged that not all publications included in our review, address the problem of endogeneity, and prices endogeneity in particular [50]. It may be that insurance coverage, major price changes as well as unobserved preferences, for example due to unmeasured attitudes, experience and culture, influence both demand and prices, and cause the inconsistency in price-elasticity estimates mentioned above. To be able to correct for this, an instrumental variable procedure and generalized method of moments could be used [66–69]. Given the above considerations, it is difficult to conclude whether the price impact in case of out-patient physician services is unambiguous and whether the variations in price-elasticity reported in different publication are genuine. This creates uncertainty regarding the relevance of the existing empirical micro-level models of demand for out-patient physician services for the evaluation of patient payment policies.

Despite the methodological limitations, the existing empirical evidence on price-elasticity of demand for out-patient physician services and its determinants (primarily income), has an important implication. They indicate that the patient payments might have certain potential to influence consumers’ decision whether to use these services, especially among low-income groups. If the demand for out-patient physician services in a given country or in a specific (income) group, is characterized by a high price elasticity, the implementation of patient payments may have a considerable effect on overall efficiency and equity in the provision of such services. For this reason, an adequate demand analysis is essential for the appropriate design of patient payments and the exemption mechanism that should accompany them [1,47,48].

Other possible determinants of demand for out-patient physician services in the publications reviewed include consumer features, time-related payments by consumers, the price of alternative health care services and quality of services provided. Although not all publications confirm the significance of all factors, their potential effects could also be relevant to policy making. For example, when implementing patient payments, health policy makers should take into account non-monetary costs for the population (e.g., waiting and travelling time) when using health care services. In case these costs are high, consumers could be discouraged from seeking out-patient physician services even when provided free-of-charge at the point of utilization [44,45]. At the same time, the location of out-patient physician services in reachable distance could make the introduction or increase of patient payments for these services more acceptable to the population.

The quality of out-patient physician services provided under patient payments also needs to be considered. In particular, monetary payments could have a less discouraging impact when they are followed by quality improvements, such as increased range of services, availability of necessary drugs, and quality of consultations [41]. Quality parameters could have an impact on price responsiveness, although authors [19,42,48] do not completely agree on the magnitude of this impact. The differences in conclusions could be attributed to diverse assumptions about quality parameters and their measurements, as well as to different attitudes within the population studied. Therefore, the importance of various quality parameters for different categories of health care users, as well as the measures of quality from the consumer perspective, need to be taken into account in further research related to the evaluation of patient payment policies.

## 5. Conclusions

It can be concluded from the review that integrated models of demand for out-patient physician services that can be used for the evaluation of patient payment policies are lacking. Hitherto, the empirical research does not offer a universal methodology for modeling demand for out-patient physician services with clear evidence on reliability and validity. The empirical research also fails to offer policy-makers a strategy for developing a country-specific model of demand for out-patient physician services suitable for the evaluation of patient payment policies in their countries. In particular, theoretically important factors (such as provider behavior, consumer attitudes, experience and culture, and informal patient payments) are not considered. Although we recognize that it is difficult to measure these factors and to incorporate them in the demand models, it is apparent that there is a gap in research necessary for the construction of an effective patient payment scheme. This gap in research can explain to a certain extent the limited number of policy analyses prior to the implementation of patient payment schemes or their subsequent amendments.

Based on the main findings in this paper, several recommendations for research and health policy-making could be outlined. To be useful for the analysis of patient payment policies, the model of consumer demand for out-patient physician services could take into account the exiting empirical analyses (e.g., [70,71]). However, it should also seek to integrate the level of informal patient payments (where relevant) and the behavior of health care providers. The model of demand should also account for the potential impact of other external factors (such as consumer attitudes, experience and culture).

Lack of empirical evidence relevant to the construction of an effective and equitable patient payment mechanism might have undesirable consequences in the health care sector. Extremely high patient payments could prevent consumers from using health care services that they really need resulting in a deterioration of population health. Alternatively, too low patient payments could fail to reduce excess demand for health care and to generate additional health care revenue. An adequate demand model could facilitate the design of patient payment mechanisms although its application would be to a great extent dependent on the vision and interests of health policy-makers.

## Figures and Tables

**Table 1.** Overall characteristics of the 46 publications included in the review.

**Characteristic of the publication**	Number of publications (%)*	**Publication reference number**

**Year of publication**		
2005–until present	11 (24)	[15,18,27,31,32,39,42–44,46,51]
2000–2004	10 (22)	[19,23,26,30,41,48,50,52,54,55]
1995–1999	7 (15)	[16,21,22,28,37,45,56]
1990–1994	4 (9)	[13,29,36,47]
1985–1989	7 (15)	[12,17,20,33,38,40,57]
1984 and before	7 (15)	[14,24,25,34,35,49,53]
Not clear	-	-

**Origin of the study**		
Africa	7 (15)	[19,27–29,41,47,48]
Asia	9 (20)	[12,15,16,22,37,42–44,46]
Europe	12 (26)	[20,21,23,31,32,45,50–55]
North America	18 (39)	[13,14,17,18,20,24–26,30,34–36,38–40,49,56,57]
South America	1 (2)	[33]
Australia and New Zealand	-	-
Not clear	-	-

**Original research setting**		
Households	24 (52)	[12,13,15,17,19,24,26,27–29,33,36,38,40,41,43–48,51,54,56]
Health care organizations	5 (11)	[12,16,36,42,53]
Other	13 (28)	[14,17,18,20,23,34–37,50,52,55,57]
Not clear	5 (11)	[22,25,32,39,49]

**Aim of the study**		
To model demand/use of physician services	11 (24)	[15,17,19,25,26,29,34,43,46,53,56]
To analyze the impact of health insurance	5 (11)	[18,21,30,32,39]
To assess the impact of patient payments	28 (61)	[13,14,16,20,22–24,27,28,31,33,35–38,40–42,44,47–52,54,55,57]
To model the choice of provider	3 (7)	[12,27,45]
Not clear	-	-

* Sum of publications in columns can be more than 46 as one article can be listed in more than one category; percentages in brackets are given for orientation purposes only (46 articles = 100%).

**Table 2.** Specificity of the data collection reported in the 46 publications included in the review.

**Characteristics of the data collection**	Number of publications (%)*	**Publication reference number**

**Study design**		
Controlled experiment	4 (9)	[13,26,38,40]
Natural experiment	6 (13)	[19–21,23,51,52]
Panel or pooled cross-section	13 (28)	[14,15,18,30,32,34–37,49,54,55]
Cross-sectional study	23 (50)	[12,16,17,22,24,25,27–29,31,33,39,41–48,53,56,57]
Not clear	-	-

**Study population**		
All consumer groups	10 (22)	[17,19,26,29,31,32,38,40,45,57]
Specific age group (e.g., children, adults)	7 (15)	[13,15,22,25,34,44,56]
Specific gender group (male, female)	2 (4)	[24,36]
Specific social group (e.g., poor, rural, insured)	14 (30)	[12,14,18,20,21,23,30,35,37,39,46,49,52,54,55]
Patients only or those with health problems	12 (26)	[16,27,28,33,34,36,41,42,44,48,53]
Not clear	1 (2)	[50]

**Sample selection**		
At random	9 (20)	[13–17,36,47,51,54]
Stratified random	10 (22)	[12,19,24,27,29,38,40,41,46,57]
Convenient sample	12 (26)	[13,20,22,28,30,35,37,44,48,49,53,55]
Specific group (e.g., workers, poor, pregnant)	3 (7)	[21,26,39]
Not clear	12 (26)	[23,25,31–34,36,43,45,50,52,56]

**Sample size**		
Less than 1000 respondents	8 (17)	[12,22,24,25,27,34,42,44]
1000–10,000 respondents	24 (52)	[13–16,18,21,28,29,32,33,36,38–41,46–50,54,56,57]
More than 10,000 respondents	12 (26)	[19,20,23,26,30,31,35,37,43,51,53,55]
Not clear	2 (4)	[17,52]

**Response rate**		
50%–70%	1 (2)	[42]
More than 70%	4 (9)	[15,24,29,38]
Not reported	41 (89)	[12–14,16–23,25–28,30–37,39–41,43–57]

**Methods of data collection**		
Face-to-face interview	7 (15)	[17,19,24,27,29,41,50]
Telephone interview	1 (2)	[50]
Patients records, administrative files, claims	19 (41)	[13–18,20,21,23,30,34–38,40,52,53,55]
Existing dataset (e.g., national surveys)	20 (43)	[12,13,22,25,26,28,33,36,39,43–47,51,54,56,57]
Questionnaire	5 (11)	[38,40–42,50]
Not clear	1 (2)	[49]

* Sum of publications in columns can be more than 46 as one article can be listed in more than one category; percentages in brackets are given for orientation purposes only (46 articles = 100%).

**Table 3.** Specificity of the analysis reported in the 46 publications included in the review.

**Characteristics of the demand modelling**	Number of publications (%)*	**Publication reference number**

**Overall approach**		
Based on revealed preferences	45 (98)	[12–41,43–57]
Based on stated preferences	1 (2)	[42]
Mixed approach	-	-
Not clear	-	-

**Nature of the dependent variable**		
Probability of visiting a physician	22 (48)	[13,15,16,19,21,24,25,29,30,32,37,39,40,42,44,45,47,49,51,53,55,57]
Number of visits to physician	22 (48)	[13,14,17,18,20,23–26,30,31,34–37,49–54,56]
Expenditure on physician visits	8 (17)	[18,30,36,39,40,54,55,57]
Number/cost of episodes of treatment	3 (7)	[13,37,38]
Type of provider chosen	13 (28)	[12,19,22,24,27–29,33,41,43,45,46,48]
Not clear	-	-

**Independent and control variables**		
Size of patient payments (direct) **	39 (85)	[12–21,23,24,26,27,29–33,35–44,47,46,49,50,52–57]
Other payments (e.g., indirect, travel, waiting)	19 (41)	[12,17–19,22,24,25,29,31,34,41–43,45–47,48,56,57]
Provider characteristics (e.g., location, quality)	17 (37)	[12,17,18,22,25,29,31,33,34,36,41–44,46,48,53]
Consumer demographic features	46 (100)	[12–57]
Consumer health status	33 (72)	[12,13,15–17,22,24–27,29–34,36,38–46,50,51,53–57]
Consumer socio-economic features	40 (87)	[12–15,17,19,20,22,23,25–27,29–34,36–57]
Consumer family features	28 (61)	[14,15,17,20–23,25,26,29,36–44,46–49,51,54–57]
Consumer insurance status	17 (37)	[12,16,24–26,29–31,34,36,37,39,46,52,54–56]
Consumer behavioral habits	5 (11)	[15,32,45,50,51]
Attitudes/perceptions	2 (4)	[24,53]
Quality of care perception	6 (13)	[25,27,41,42,48,54]
Availability of informal providers	1 (2)	[15]
Prices of other goods (e.g., food, services)	1 (2)	[43]
Not clear	1 (2)	[28]

**Stages in the modelling process**		
One stage modelling	28 (61)	[12–16,18,21–25,27,28,31–33,36,37,41–44,46,47,50,52,53,56]
Multiple stages modelling	18 (39)	[19,26,29,30,36,39,40,45,48,51,54,55,57]
Not clear	-	-

**Analytical model**		
Least squares (OLS/2SLS/GLS/RE/FE/DiD)	18 (39)	[13,14,18,20,23,25,30,34,36–38,40,49,52–55,57]
Multinomial/Nested/Conditional Logit	13 (28)	[12,19,22,24,27–29,33,41,43,45,46,48]
Binary Logit/Probit	15 (33)	[13,15,21,25,30,36,37,39,40,44,47,51,53,55,57]
Tobit	2 (4)	[24,53]
Count data model	7 (15)	[17,26,31,50,51,54,56]
Duration model	3 (7)	[15,16,42]
ANOVA, ACONOVA	2 (4)	[35,40]
GLM	2 (4)	[39,55]
GMM	1 (2)	[50]
Non-parametric estimation	1 (2)	[32]
Not clear	-	-

**Other statistical methods**		
Simulation methods	13 (28)	[16,19,26,29–31,33,38,41,43–45,48]
Descriptive statistics	32 (70)	[12,13,15–20,22,23,25,27,29–31,32,36–39,41,42,44,46,48,49,51–55,57]
Chi-square, T or F-test	11 (24)	[20,21,23,26,27,35,39,40,45,51,52]
Not reported	5 (11)	[14,28,34,47,56]

**Goodness-of-fit statistics**		
Reported	33 (72)	[13–16,18,19,23–27,29,31,34–38,41,42,44–49,51–55,57]
Not reported	13 (28)	[12,17,20–22,28,30,32,33,39,40,43,56]

* Sum of publications in columns can be more than 46 as one article can be listed in more than one category; percentages in brackets are provided for orientation purposes only (46 articles = 100%);

** Several articles included in the review used indirect specification of the user fees.

**Table 4.** Major findings reported in the 46 publications included in the analysis.

**Summary of major findings**	Number of publications (%)*	**Publication reference number**

**Changes in quantity demanded due to changes in price (*i.e.*, patient payment)**		
Significant in general	30 (65)	[13,16,18,20,22,27–35,36,38,40,41,43,44,46–49,52–57]
Significant for certain parameters or groups	6 (13)	[19,23–25,37,39]
Statistically insignificant	4 (9)	[12,15,21,51]
Not clear	6 (13)	[14,17,26,42,45,50]
Not reported	-	-

**Price elasticity of demand (average value reported)**		
Less than 0.10	8 (17)	[15,17,18,23,24,32,34,54]
0.10–0.50	16 (35)	[16,25,26,28,31,34,36,38–40,43,44,46,52,55–57]
0.51–0.99	3 (7)	[33,47,48]
1 or higher	2 (4)	[22,47]
Not clear	7 (15)	[13,14,19,20,27,35,41]
Not reported	10 (22)	[12,21,29,30,37,42,45,49,50,53]

**Significant determinants of price elasticity of demand**		
Consumer income	18 (39)	[14,19,22,23,25,27,33,34,36,40,42–48,52]
Consumer demographic features	5 (11)	[20,23,42,47,53]
Consumer health status	5 (11)	[16,20,38,42,57]
Opportunity costs, time	1 (2)	[45]
Provider characteristics (e.g., services, quality)	14 (30)	[13,16,19,20,25,27,31,34,41,42,45,46,54,55]
Insurance status (type of coverage)	2 (4)	[39,54]
Availability/price of other services/goods	8 (17)	[18,23,31,33,35,43,45,48]
Magnitude of service price	3 (7)	[26,42,54]
Not reported	12 (26)	[12,17,21,26,28–30,32,37,50,51,53,56]

**Significant determinants of demand other than price (*i.e.*, other than patient payments)**		
Consumer income	15 (33)	[12–15,19,24,25,27,29,34,36,40,46,54,57]
Consumer demographic features	26 (57)	[13,15–17,22,25,27,29,31,35,37–39,41–49,50,53,55–57]
Consumer social features	11 (24)	[15,22,25,29,31,33,37,53,54,56,57]
Consumer health status	21 (46)	[12,15–17,24,27,29,30,36,38,39,43–46,50,51,53–57]
Consumer family features	9 (20)	[22,25,27,39,41,47,54,56,57]
Consumer insurance status	6 (13)	[16,29,36,39,50,59]
Payments for other health care services	2 (4)	[12,18]
Non-monetary expenses (opportunity costs)	12 (26)	[17,22,24,31,33,42–46,48,56,57]
Provider characteristics (e.g., services, quality)	11 (24)	[17,22,25,29,31,34,41–43,48,53,54]
Availability of other health care services	2 (4)	[15,45]
Not reported	7 (15)	[20,21,23,26,28,32,52]

**Validity, reliability and generalizability of the results**		
Reliability is clear	19 (41)	[13,15,18,23,29–31,38–40,46,47,48,50,52–55,57]
Reliability is uncertain	22 (48)	[12,14,16,17,19–21,24–27,30,32–37,41,42,44,45,51]
Reliability is not analyzed	5 (11)	[22,28,43,49,56]
Validity is clear	17 (37)	[15,16,23,26,27,31,36,38,41,42,46,47,51,52,54,55,57]
Validity is uncertain	29 (63)	[12–14,17–22,24,25,28–30,32–35,37,39,40,43–45,47–49,50,53,56]
Validity is not analyzed	-	-
Generalizability is clear	7 (15)	[12,16,29,38,40,42,57]
Genelizability is uncertain	19 (41)	[14,15,18,21–24,27,28,36,37,39,41,46,47,51,52,55]
Generalizablity is not analyzed	20 (43)	[13,17,19,20,25,26,30–35,43–45,48–50,53,54,56]

* Sum of publications in columns can be more than 46 as one article can be listed in more than one category; percentages in brackets are given for orientation purposes only (46 articles = 100%).
